# The influence of solid/liquid separation techniques on the sugar yield in two-step dilute acid hydrolysis of softwood followed by enzymatic hydrolysis

**DOI:** 10.1186/1754-6834-2-6

**Published:** 2009-03-16

**Authors:** Sanam Monavari, Mats Galbe, Guido Zacchi

**Affiliations:** 1 Department of Chemical Engineering, Lund University, SE-221 00 Lund, Sweden

## Abstract

**Background:**

Two-step dilute acid hydrolysis of softwood, either as a stand-alone process or as pretreatment before enzymatic hydrolysis, is considered to result in higher sugar yields than one-step acid hydrolysis. However, this requires removal of the liquid between the two steps. In an industrial process, filtration and washing of the material between the two steps is difficult, as it should be performed at high pressure to reduce energy demand. Moreover, the application of pressure leads to more compact solids, which may affect subsequent processing steps. This study was carried out to investigate the influence of pressing the biomass, in combination with the effects of not washing the material, on the sugar yield obtained from two-step dilute acid hydrolysis, with and without subsequent enzymatic digestion of the solids.

**Results:**

Washing the material between the two acid hydrolysis steps, followed by enzymatic digestion, resulted in recovery of 96% of the mannose and 81% of the glucose (% of the theoretical) in the liquid fraction, regardless of the choice of dewatering method (pressing or vacuum filtration). Not washing the solids between the two acid hydrolysis steps led to elevated acidity of the remaining solids during the second hydrolysis step, which resulted in lower yields of mannose, 85% and 74% of the theoretical, for the pressed and vacuum-filtered slurry, respectively, due to sugar degradation. However, this increase in acidity resulted in a higher glucose yield (94.2%) from pressed slurry than from filtered slurry (77.6%).

**Conclusion:**

Pressing the washed material between the two acid hydrolysis steps had no significant negative effect on the sugar yields of the second acid hydrolysis step or on enzymatic hydrolysis. Not washing the material resulted in a harsher second acid hydrolysis step, which caused greater degradation of the sugars during subsequent acid hydrolysis of the solids, particularly in case of the vacuum-filtered solids. However, pressing in combination with not washing the material between the two steps enhanced the sugar yield of the enzymatic digestion step. Hence, it is suggested that the unwashed slurry be pressed to as high a dry matter content as possible between the two acid hydrolysis stages in order to achieve high final sugar yields.

## Background

Interest in ethanol as an alternative fuel is increasing. Bioethanol is not only an attractive substitute for oil, but it also has positive effects on the environment, such as low net carbon dioxide emission. Unlike fossil fuels, ethanol is a renewable energy source, and can be produced from lignocellulosic biomass. Lignocellulosic materials are an attractive feedstock because they are available in large quantities [1,2]. In Sweden, Canada and parts of the USA softwood (pine and spruce) may become the major renewable source of ethanol production, mitigating some of the environmental impacts of petroleum-based fuels [3-7].

In order to achieve high yields and high ethanol concentrations when producing ethanol from lignocellulosics through enzymatic hydrolysis and fermentation, the cellulosic biomass must first be pretreated. Pretreatment, which is a crucial step for the enzymatic digestibility of the biomass, is one of the most expensive process steps. Various technologies are currently being used for the conversion of the cellulose and hemicellulose in biomass into fermentable sugars, including hot water or acid hydrolysis, steam explosion, ammonia fibre explosion (AFEX) and alkaline hydrolysis [8-10]. However, previous studies have shown that dilute acid steam pretreatment is considered to be one of the most promising methods of pretreatment for softwood [11,12], particularly when SO_2 _is used as the impregnating agent [6,13,14]. It has been proven that more severe conditions are required during steam pretreatment to improve the digestibility of cellulose, but this will also cause greater degradation of the hemicellulose sugars [15-17]. To overcome this, two-step dilute acid pretreatment with separation and washing of the solid residue between the two steps has been proposed [15,18-22]. Running the first step at lower severity (mainly lower temperature) and the second step at higher severity (higher temperature) results in increased hemicellulose recovery and improved enzymatic digestibility of the cellulose from the recalcitrant structure of lignocellulosics. The same concept has been proposed for the two-stage acid hydrolysis process, that is without enzymatic hydrolysis, to maximize the hemicellulose sugar yield [23]. This concept is also being used in the Swedish pilot plant in Örnsköldsvik, SEKAB [24], where two-stage acid hydrolysis is used either as a stand-alone hydrolysis process or as a pretreatment for the enzymatic hydrolysis process. In both cases, most of the hemicellulose is hydrolyzed in the first step and then recovered in the liquid fraction by filtration or pressing and washing of the solid material. The second step is then performed at higher severity to open up the structure of the cellulose and make it more accessible to enzymes, in the case of the enzymatic process, or to hydrolyze the cellulose in the case of the acid hydrolysis process.

In an industrial process it is desirable to maintain the pressure between the two acid hydrolysis steps to avoid energy losses from steam flashing, requiring that the material be reheated to a high temperature between the steps [25]. This is possible through mechanical pressing of the pretreated slurry at high temperature and vapour pressure. Pressing the slurry at high pressure, after both the acid hydrolysis and the washing steps, makes the material more compact. The mechanical compression forces applied to the pretreated material could cause deterioration of the wood matrix and thus influence the extent of diffusion of the catalyst during impregnation, as well as the heat transfer to the woody material [23,26]. Mechanical dewatering itself is an energy-demanding process, but it helps to achieve a higher total solids content, and thus reduces the steam demand in the second step [23]. In addition, thorough washing of the pretreated solids at high pressure with good efficiency, to recover most of the water-soluble compounds, is technically challenging, and may also decrease the concentration of soluble sugars through dilution.

In the current study, the influence of pressing the slurry on the sugar yield in two-step dilute acid hydrolysis of spruce with SO_2 _impregnation was investigated. A study was also performed on the effect of washing with and without pressing of the material between the two acid hydrolysis steps. The conditions for the second acid hydrolysis step were chosen so as to be suitable for pretreatment, that is, for the enzymatic process. However, the results obtained regarding the effects of pressing and washing would be applicable to the pure acid hydrolysis process. The first step would be the same, while the second step in a pure acid hydrolysis process would be performed at a higher severity than that investigated in this study to solubilize a larger fraction of the cellulose. The effects of pressing and washing were assessed by determining the content of carbohydrates after the first and second acid hydrolysis steps. For the enzymatic process, the results of the pretreatment experiments were also assessed by enzymatic hydrolysis of the washed water-insoluble solids (WIS), with and without pressing of the slurry after the second acid hydrolysis step, to determine the maximum fermentable sugar yield in the enzymatic hydrolysis process.

## Methods

**Figure 1:**
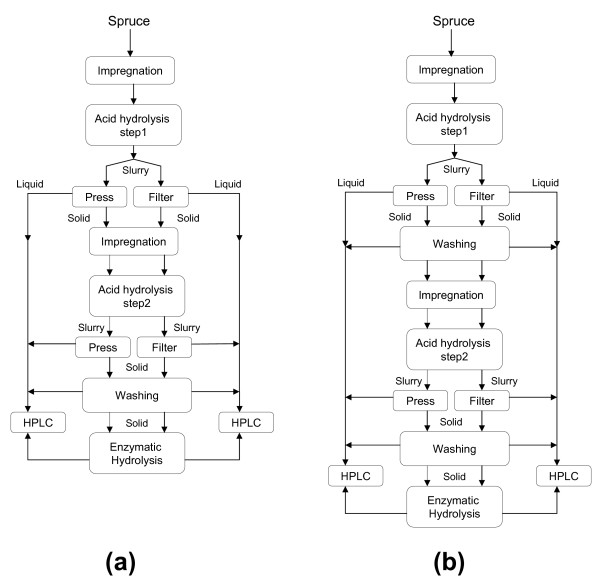
**Schematic illustration of the experimental designs for the evaluation of different acid hydrolysis processes**. a) Without washing the solids, (using Material I as feedstock), and b) with washing of the solids between the two acid hydrolysis steps and (using Material II).

A number of different procedures concerning the dewatering of the pretreated slurry with and without washing of the solids were investigated in the current study. The experimental designs are schematically illustrated in Figure 1. The softwood was first milled, then impregnated with SO_2 _and subjected to the first acid hydrolysis step in a steam-pretreatment unit using high-temperature steam. The resulting material was separated into two fractions; one fraction was pressed and the other was vacuum filtered to separate the liquid and solid residues. The resulting liquid was analyzed regarding its sugar content. The solid fraction was then re-impregnated with SO_2 _and steam pretreated again (second acid hydrolysis step). The liquid obtained was analyzed regarding its sugar content. The solid residue from the second step was washed and subjected to enzymatic hydrolysis to determine the overall sugar yield of the enzymatic process.

### Raw materials

Fresh-chipped spruce, *Picea abies*, free from bark was kindly provided by Widtsköfle Sawmill (Degeberga, Kristianstad, Sweden). The wood chips were re-chipped and chips between 2 and 10 mm were used. The fresh raw material was stored in plastic bags at 4°C. Material I (50%DM) was used in experiments without washing between two steps (Figure 1a) while material II (38% DM) was utilized in the experiments with washing the solids (Figure 1b). The composition of the raw material, in terms of carbohydrates, lignin and ash, was determined according to the standardized methods of the National Renewable Energy Laboratory (NREL) [27].

### First acid hydrolysis step

Prior to pretreatment, that is dilute acid hydrolysis, the chips were impregnated with 3% SO_2 _(w/w based on the water content of the wood chips). Pretreatment was carried out in a 10-litre reactor which has been described previously [28]. The conditions for the first acid hydrolysis step have been optimized previously for fresh spruce chips and found to be 190°C and 2 min [15,25]. Several batches of the pretreated material were produced before emptying the flash chamber. The pretreated slurry was then mixed, (Materials I and II separately) and each divided in two identical fractions. One fraction was pressed with a hydraulic press, at a pressure corresponding to 16 bar on the slurry, to separate the solids and liquids. The other fraction was filtered through an ordinary Buchner vacuum filter. In one series of experiments, the resulting solid fraction was washed twice with excess hot water, approximately three times the volume of the liquid present in the solid material, to remove the rest of the water-soluble compounds remaining inside the solid cake (Figure 1). The washed cake was then pressed or vacuum filtered. The liquid fractions and the washing water were then analyzed with respect to the concentration of water-soluble compounds.

### Second acid hydrolysis step

The resulting solids from step one from both experimental series (Figure 1) were impregnated again with 3% SO_2 _(w/w) and steam pretreated separately at 210°C for 5 min, which had been found to be the optimal conditions in previous studies [15,25]. The pretreated slurry was pressed or vacuum filtered to separate the liquid and the solids. The liquid fractions were analyzed regarding soluble sugars and degradation products, and the solid fractions were subjected to enzymatic hydrolysis.

### Enzymatic hydrolysis

Enzymatic hydrolysis was performed on the washed solids from the second acid hydrolysis step to assay the maximum digestibility of the wood chips, using enzymes. This was done to investigate the efficiency of the pretreatment regarding the choice of separation technique [15,25]. The enzymes used in this study were Celluclast 1.5 L (65 FPU/g and 17-β-glucosidase IU/g enzyme solution) and the β-glucosidase preparation Novozyme 188 (376β-glucosidase IU/g enzyme solution) kindly provided by Novozyme A/S (Bagsvaerd, Denmark). The solids from the second step were washed with hot water. The pretreated solids that had been pressed in previous step were pressed once more after washing to remove the water-soluble compounds, while filtered material was vacuum filtered again. Enzymatic hydrolysis was performed on 2% WIS (w/w) to reduce end-product inhibition. Ten grams washed solids together with Celluclast 1.5 L (15 FPU/g WIS; 33.5 and 33 FPU/g glucan, respectively for material I and II) and Novozyme 188 (18 IU/g WIS; 39.6 and 40.2 IU/g glucan, respectively for material I and II) were immersed in 0.1 ml/l sodium acetate buffer (pH 4.8) to a total mass of 500 g under non-sterile conditions. In order to prevent the growth of microorganisms, 0.2 g NaN_3 _was added to each hydrolysis batch. Enzymatic hydrolysis was performed at 40°C for 96 h. Samples were withdrawn after 0, 2, 4, 6, 8, 24, 48, 72, and 96 hours to monitor the progress of hydrolysis.

### Analysis

The amounts of sugars (monosaccharides) and inhibitors were determined using high performance liquid chromatography (HPLC) (Shimadzu, Kyoto, Japan) equipped with a refractive index detector (Shimadzu). The total sugars present in the pretreatment filtrate were analyzed according to the total-sugar analysis method of the NREL [29] using dilute acid hydrolysis of the filtrate. The content of oligosaccharides was calculated as the difference between the monosaccharide content measured before and after hydrolysis. The compositions of the raw materials and pretreated solids were evaluated using the NREL procedure for determination of structural carbohydrates and lignin in biomass [27]. All the samples were diluted and passed through a 0.2 μm filter before HPLC analysis. Acidic samples were neutralized by the addition of CaCO_3 _before analysis. Glucose, xylose, galactose, arabinose and mannose were separated on an Aminex HPX-87P column (Bio-Rad, Hercules, CA, USA) run at a flow rate of 0.5 ml/min at 85°C, with water as the eluent. Acetic acid, ethanol, lactic acid, 5-hydroxymethylfurfural (HMF) and furfural were separated by means of an Aminex HPX-87H column (Bio-Rad) at 65°C, using 0.5 ml/min 5 mM H_2_SO_4 _as eluent.

## Results and discussion

### Raw material

The composition of the raw materials is given in Table 1. The contents of carbohydrates, lignin and the remaining components were found to be within the range found in the literature. Glucan and mannan constitute more than 55% of the sugars in fresh spruce, representing the two main fermentable carbohydrates of the raw material [14,30].

**Table 1:** Composition of spruce, expressed as % dry matter

**Composition**	**Dry matter (%)** **Material (I)**	**Dry matter (%)** **Material (II)**
Glucan	45.4	44.8
Xylan	3.8	6.0
Galactan	3.2	2.5
Arabinan	1.3	1.3
Mannan	10.3	13.8
Total lignin	31.5	28.9
Other components^2^	4.5	2.7

^1 ^Total lignin is the sum of the acid-soluble and insoluble lignin.

^2 ^Other components are extractives, ash, proteins, and some unknown compounds.

### Dilute acid hydrolysis

Two different experimental setups were performed in this study. One of the objectives was to study the influence of separation method between the two acid hydrolysis steps through assessment of the sugar yield. The other objective was to investigate the effect of not washing the pressed material between the two steps on the sugar yields in the two-step dilute acid process (See Figure 1).

All the sugar yields are expressed as percentages of that theoretically available in the raw material, unless otherwise stated. The pentose content is not reported due to the low concentrations of pentoses in spruce. The composition of the solid materials after the first and second acid hydrolysis steps is given in Table 2.

**Table 2:** Composition of the pretreated material after the first and second acid hydrolysis steps, expressed as % of dry matter

**Pretreatment^1^**	**Glucan**	**Xylan**	**Galactan**	**Arabinan**	**Mannan**	**Lignin** **(acid-soluble)**	**Lignin** **(insoluble)**
First step, +W	52.7	2.1	1.0	0.7	2.0	0.3	37.8
First step, -W	58.7	0.0	1.9	0.0	1.5	0.7	38.3
Second step, P+W	56.6	0.0	0.9	0.7	1.6	0.3	40.9
Second step, F+W	57.8	0.0	0.9	0.8	1.5	0.3	39.8
Second step, P-W	54.8	0.0	1.8	0.0	1.3	0.6	42.5
Second step, F-W	53.9	0.0	1.6	0.0	1.3	0.6	43.8

^1 ^Experimental designs: with washing (+W) and without washing (-W) of pressed (P) and vacuum-filtered (F) material.

### Effects of pressing of the slurry

**Figure 2:**
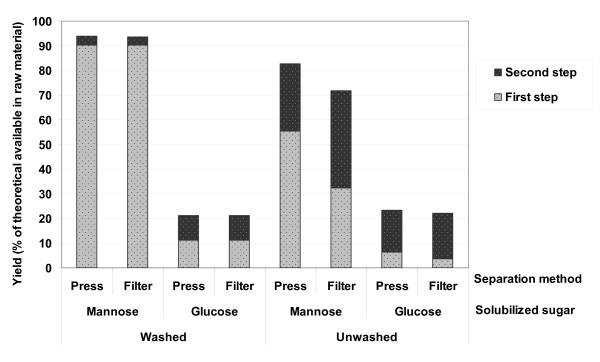
**The yield of solubilized sugars (glucose and mannose) after two-step dilute acid hydrolysis with different dewatering methods and the effect of washing the slurry between the two acid hydrolysis steps**.

Figure 2 shows the overall amount of mannose and glucose solubilized after two-step dilute acid hydrolysis with washing of the solids between the two hydrolysis steps, together with the results from the experiments without washing. When the material was washed between the two steps most of the mannose was solubilized and was recovered after the first step, whereas the major part of the glucose was still present as glucan in the solid material. The recovery of mannose and glucose in the WIS after each acid hydrolysis step is shown in Table 3. These results indicate that, regardless of the choice of dewatering method (vacuum filtration or pressing), over 90% of the mannose was removed from the cake by thorough washing.

**Table 3:** Recovery of the glucose and mannose in the water-insoluble solids after the first and second acid hydrolysis steps, expressed as % of the theoretical available in the raw material

**Pretreatment^1^**	**Glucose**	**Mannose**
First step, +W	86.6	10.0
First step, -W	86.0	9.8
Second step, P+W	70.7	6.3
Second step, F+W	71.2	5.9
Second step, P-W	69.6	7.0
Second step, F-W	65.4	6.8

^1 ^Experimental designs: with washing (+W) and without washing (-W) of pressed (P) and vacuum-filtered (F) material.

The remaining mannose and some of the glucose were solubilized in the second dilute acid hydrolysis step. Since most of the mannose had already been solubilized in the first step, the mannose yield increased by only about 3%. The low glucose yield, 10%, is due to the severity of the conditions applied for the second acid hydrolysis step. The conditions used for the second step were chosen so as to be optimal for the enzymatic hydrolysis step, that is, to obtain the maximum digestibility of the cellulose [15]. Running an acid hydrolysis process only would require more severe conditions, which would result in greater solubilization of glucose during the second step. The yields obtained indicate that when the material was washed between the two acid hydrolysis steps, pressing of the slurry up to 16 bar had no significant impact on the soluble sugar yields, compared with vacuum filtration.

### Effects of not washing of the slurry

Not washing the pretreated slurry between the two acid hydrolysis steps led to a lower hemicellulose yield (see Figure 2). The mannose yield from the first acid hydrolysis of the pressed material is higher than that of the filtered material, 55.6 and 32.4% (of the theoretical), respectively. This is due to the removal of a higher volume of liquid containing water-soluble compounds from the pretreated slurry for the pressed material. However, the amount of mannose released in the slurry after the first step was 87.4% for both pressed and vacuum-filtered material. This means that a large fraction of soluble sugars remained in the slurry that was fed to the second hydrolysis step.

The solids from step one were re-impregnated and then steam pretreated at 210°C for 5 min. The rather high acid content (pH 1.8) of the slurry together with the re-impregnation with SO_2 _%(w/w) increased the acidity of the material. This, together with the more severe hydrolysis conditions (higher temperature) applied in the second step, resulted in an even higher severity. This enhanced the solubilization of the carbohydrates but at the same time led to the formation of degradation products, and therefore resulted in lower yields. The severity was higher for the vacuum-filtered material since it contained more liquid, and thus more acid, when it was re-impregnated, whereas the pressed solids contained a smaller amount of liquid. The higher severity, together with the higher amount of soluble sugars in the liquid from the first step for vacuum-filtered material, resulted in higher sugar degradation.

Figure 2 shows that not washing the pretreated solids between the two hydrolysis steps led to overall glucose yields similar to those in the washed material. Higher first-step glucose yields in the washed solids are due to the complete removal of solubilized sugars whereas, as discussed above, partial recovery of sugars from the unwashed material after the first step resulted in a higher glucose yield in the second acid hydrolysis step.

**Figure 3:**
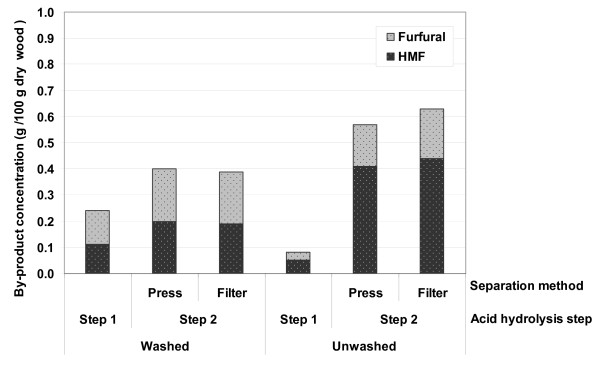
**Concentration of sugar degradation products in the liquid obtained from the second acid hydrolysis step (g/100 g raw material) used in the process**.

Further degradation of hexoses and pentoses in an acidic environment at high temperature and pressure results in the formation of by-products, mainly HMF and furfural. Figure 3 shows the concentration of sugar-degradation products, per 100 g raw material, formed during the two acid hydrolysis steps in both series of experiments. The lower severity of the first step resulted in the formation of smaller amounts of by-products. The small differences in the first step between the two experimental setups are mainly due to the differences in the composition of the raw material used. However, the concentration of the sugar degradation products increased in the second step for both experimental setups. The total amount of HMF and furfural produced in the second step, per 100 g fresh spruce, was 0.40 g for washed material, both pressed and filtered, and 0.57 and 0.63 (g/100 g raw material), for unwashed, pressed and vacuum-filtered solids, respectively. The increase in the amount of inhibitors formed in the unwashed material during the second step is due to the elevated acidity of the remaining liquid from the first step, as described above. Moreover, under harsh conditions HMF and furfural can be further degraded to other by-products, such as formic acid and levulinic acid, which were not quantified in the current study.

### Enzymatic hydrolysis

**Figure 4:**
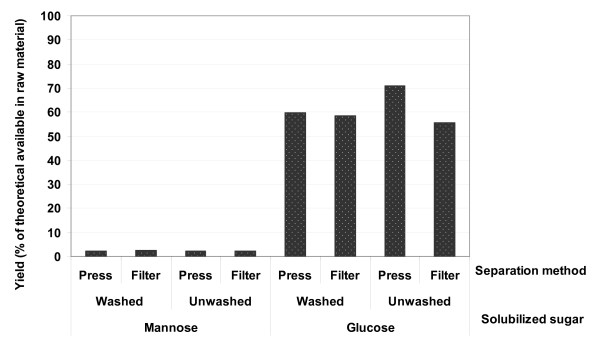
**Yield of fermentable sugars during enzymatic hydrolysis of washed solids from the acid hydrolysis step for different process configurations regarding the choice of separation of solid and liquid and the effect of the washing the slurry between the two acid hydrolysis steps**.

Enzymatic hydrolysis was carried out on washed insoluble solids from the second acid hydrolysis step to assess the impact of pressing, with and without washing of the material between the two acid hydrolysis steps, on the digestibility of the solids and thus the sugar yields. Figure 4 shows that most of the glucose was solubilized during enzymatic hydrolysis. When the pretreated slurry was washed between the two acid hydrolysis steps the glucose yield was about the same for pressed and vacuum-filtered material, 59% ± 1.5% (of that theoretically available in the raw material). The lowest conversion of cellulose (55%) was obtained from the unwashed, filtered material owing to the high acidity of the solids during the second acid hydrolysis step, resulting in degradation of sugars. However, not washing the pressed material before the second step resulted in the maximum hydrolysis of cellulose when using enzymes (71%). The slightly elevated severity of the second acid hydrolysis step in this case improved the accessibility of the cellulose chain to enzymatic attack.

### Overall sugar yield

The overall yield of fermentable sugars, glucose and mannose, is presented in Table 4. Washing the material between the two acid hydrolysis steps, followed by enzymatic hydrolysis of the WIS, resulted in comparable yields for pressed and vacuum-filtered material, 86 and 84%, respectively. This is due to complete removal of the water solubles from the slurry after the first acid hydrolysis step resulting in more or less the same pretreatment in the second step. The overall sugar yield for the unwashed solids reached 93 and 77.5%, respectively for pressed and vacuum-filtered material. The higher severity of the second acid hydrolysis step, as explained earlier, resulted in both improved digestibility and increased degradation of the sugars in the liquid from the first step. For the filtered material, with more liquid available from the first step, this resulted in a somewhat lower total sugar yield than that obtained for washed material while for the pressed material, containing less liquid, it resulted in a higher total sugar yield compared with washed material.

**Table 4:** Overall yield of fermentable sugars, glucose and mannose, for different process configurations in the two-step dilute acid hydrolysis process followed by enzymatic hydrolysis, expressed as % of the theoretical available in the raw material

**Pretreatment^1^**	**Pressed + W**	**Filtered + W**	**Pressed - W**	**Filtered - W**
Yield (Glucose + Mannose)	86.0	84.0	93.0	77.5

^1 ^Experimental designs: with washing (+W) and without washing (-W)

## Conclusion

Two-step acid hydrolysis of spruce chips with 3% SO_2 _produced an easily digestible material resulting in high yields of fermentable sugars (mannose and glucose) during enzymatic hydrolysis. The results obtained in the current study demonstrate that pressing the slurry with a hydraulic press (up to 16 bar) after the first step had no significant negative effect on the sugar yields from a dilute acid process or enzymatic hydrolysis, if the material was washed between the two acid hydrolysis steps.

Dilute acid hydrolysis of woody material in two steps with separation and washing of the pretreated solids between the steps prevented a large fraction of the solubilized sugars from being further degraded, and thus resulted in a higher overall mannose yield, which is consistent with the results obtained by Söderström et al [25]. In a process for the production of ethanol from lignocellulosic material it is highly desirable to minimize the addition of fresh water, to maintain high concentrations of the sugars and also to avoid large waste water streams [31]. Elimination of the washing step between two acid hydrolysis steps, for the filtered (not-pressed) material, led to a more severe second acid hydrolysis step. This higher severity resulted in the formation of higher amounts of sugar degradation products and, accordingly, lower sugar yields from the second acid hydrolysis step and the subsequent enzymatic hydrolysis of the solid residues.

The current study also showed that pressing without washing resulted in higher enzymatic digestibility of the pretreated material as a result of the partial recovery of the water-soluble compounds before the second pretreatment step and the consequent improved severity of the second pretreatment. Therefore, in order to achieve high sugar yields in a dilute acid process without washing the material between the steps, it is suggested that pressing to as high a dry matter as possible be applied between the acid hydrolysis steps. Nevertheless, it should be taken into consideration that according to Kim et al pressing at high pressures (41.4 bar) could alter the wood structure and lower the yields [23].

## Competing interests

The authors declare that they have no competing interests.

## Authors' contributions

SM carried out the steam pretreatment, acid hydrolysis and enzymatic hydrolysis, analyzed the results and wrote the paper. MG helped to coordinate the study and draft the manuscript. GZ participated in the design and coordination of the study and helped to draft the manuscript. All authors read and approved the final manuscript.
